# The Neural Response to Maternal Stimuli: An ERP Study

**DOI:** 10.1371/journal.pone.0111391

**Published:** 2014-11-06

**Authors:** Lili Wu, Ruolei Gu, Huajian Cai, Yu L. L. Luo, Jianxin Zhang

**Affiliations:** 1 Key Laboratory of Mental Health, Institute of Psychology, Chinese Academy of Sciences, Beijing, China; 2 Key Laboratory of Behavioral Science, Institute of Psychology, Chinese Academy of Sciences, Beijing, China; Center for BrainHealth, University of Texas at Dallas, United States of America

## Abstract

Mothers are important to all humans. Research has established that maternal information affects individuals' cognition, emotion, and behavior. We measured event-related potentials (ERPs) to examine attentional and evaluative processing of maternal stimuli while participants completed a Go/No-go Association Task that paired *mother* or *others* words with *good* or *bad* evaluative words. Behavioral data showed that participants responded faster to *mother* words paired with *good* than the *mother* words paired with *bad* but showed no difference in response to these *others* across conditions, reflecting a positive evaluation of mother. ERPs showed larger P200 and N200 in response to *mother* than in response to *others*, suggesting that *mother* attracted more attention than *others*. In the subsequent time window, *mother* in the *mother + bad* condition elicited a later and larger late positive potential (LPP) than it did in the *mother + good* condition, but this was not true for *others*, also suggesting a positive evaluation of mother. These results suggest that people differentiate *mother* from *others* during initial attentional stage, and evaluative *mother* positively during later stage.

## Introduction

The mother is important for human beings throughout their lifetime. A mother's physiological and mental state during the pregnancy contributes substantially to her child's physical and psychological health in both early and late infancy (e.g., [1]–[4]). In early childhood, the mother regulates the physiological state and behaviors of her child [5]. Attachment to their mothers is the start of children's socialization, which facilitates social and affective development [6]. Later, mothers' parenting behavior affects adolescents' emotional regulation ability and problem behaviors [7], [8]. Finally, for adults, mothers are still an essential part of individuals' attachment networks [9]. Furthermore, the perceived quality of the parent-child relationship during childhood predicts adults' psychological and physical well-being throughout the lifespan [10], [11]. In short, the significance of mothers is difficult to overestimate.

Given this physical and psychological significance, a large number of empirical studies have focused on how stimuli related to the mother, such as a mother's face or name, affect information processing. Findings from studies using behavioral (e.g., [12]–[14]), electrophysiological (e.g., [15]) and neuroimaging measures (e.g., [16]) show that maternal stimuli affect people's attention and evaluative responses. From the attentional perspective, individuals show a tendency to attend more to maternal stimuli than other kinds of stimuli across different ages. For instance, newborns (4 hours to 72 hours after birth) stare at their mother's face longer than at a stranger's face [12]–[14], [17], [18]. Furthermore, an electrophysiological study showed that mothers' faces elicited a larger negative component (Nc, occurring 400 to 800 ms, indexing attentional response) compared with a stranger's face, indicating that infants allocate increased attention to the mother's face [15]. For adults, the faces of mothers elicited greater activation in facial-specific regions than the faces of others, including strangers, celebrities or even fathers [16]. From an evaluative perspective, maternal stimuli are perceived more positively than other stimuli. For instance, people favor their mother's name [19], show a higher retrieval rate for favorable traits than for unfavorable traits after a mother-reference task [20], and are inclined to interpret their mother's neutral faces as cheerful faces [21].

Although these studies highlight attentional preference and evaluative positivity when processing maternal stimuli, two issues still remain unclear. First, the attentional bias to maternal information usually has been attributed to familiarity [e.g., 15, 16], but a recent study showed that the preference for maternal information was strongly affected by the intense attachment to mother, rather than just reflecting familiarity [21]. However, it is difficult to rule out the familiarity effect because previous studies on the mother/stranger distinction used pictures of mothers and strangers as material. Thus, effectively controlling for the familiarity of material is necessary when evaluating whether the preference for mother is due to the intense attachment. Second, previous studies have found that individuals have positive evaluations of their mothers [19], [20], [21], but this tendency might be affected by a social desirability bias, influencing participants to show favoritism to their mother over others. However, combining an implicit task with measurements of neural responses that index online mental operations independent of behavioral response processes would reduce the impact of social desirability bias on maternal evaluations.

The present study sought to examine the attentional and evaluative processing of maternal stimuli using event-related potentials (ERPs). ERPs are particularly well-suited for examining these two processes, because the ERP waveform, measured in response to a stimulus, contains a number of components that are temporally linked to the emergence of different mental operations, including perception, attention, and evaluation [22]. This property of the ERP technique makes it particularly useful for examining attentional and evaluative processes by assessing different components.

Previous research used various types of maternal stimuli, including faces, names, and basic word descriptions (e.g., “mother”) [15], [16], [19], [20]. In the present study, we used words for *mother* and *others* as stimuli. We used words instead of pictures as maternal stimuli because the evaluative dimensions in the task were described by words (good or bad). This effectively eliminated the potential effect of switching between word and picture processing during the experiment. Most importantly, words are more effective for controlling for the familiarity, because the familiarity of word stimuli could be indexed by objective measures such as frequency.

We selected the Go/No-go Association Task (GNAT, [23]) because of its suitability for ERP studies (e.g., [24], [25]). More importantly, this paradigm is suitable for examining the attentional and evaluative processing of maternal stimuli by creating contrasts between different experimental blocks. In particular, we used two blocks to explore the processing of maternal stimuli (*mother + good* and *mother + bad*) and two further blocks to explore the processing of non-specific *others* (*others + good* and *others + bad*). For all four blocks, we focused on the behavioral and neural responses to *mother* and *others* stimuli and compared them between the different conditions to test for difference in attentional and evaluative processes. The contrast between *mother* blocks and *others* blocks allowed for examining differences in attentional resource allocation between *mother* and *others*. Furthermore, the evaluative processing of maternal stimuli was examined by contrasting the *mother + good* and *mother + bad* conditions, where the evaluation of *mother* was operationalized as the associative strength of maternal stimuli with positive or negative attributes in the GNATs.

To examine the attentional processing of maternal stimuli, two ERP components, the P200 and N200 were measured and analyzed. Enhanced amplitudes of these two components are assumed to reflect increased attention to information with intrinsic personal relevance, such as self-related information [26], [27], [28]–[31]. Thus, we assessed whether participants differentiated *mother* from non-specific *others* at an early attentional stage by examining the amplitudes of these two ERP components across the four conditions. Specifically, we hypothesized that *mother* would draw more attention than *others*, as indicated by increased P200 and N200 components, because of the mother's critical role in a child's life.

In addition, to further explore the evaluative processing of maternal stimuli, the late positive potential (LPP) that is generally found to be maximal around the posterior region of the scalp (for reviews, see [32]–[35]) was examined. This component has been associated with evaluative processing [36]–[39]. Its peak latency could be used as a neural indicator of the speed of categorization and evaluation (for a review, see [33]). Inconsistency between the current stimulus representation and a previous expectation or evaluation enhances LPP amplitude [36], [37]. In the area of social cognition, the LPP has been used to assess the evaluation of specific social groups. For instance, the LPP amplitude is larger for counter-stereotype associations than stereotype-consistent associations, reflecting a violation of a previously established evaluation and implicit racial attitude [40], [41]. Meanwhile, compared with stereotype-consistent information, the LPP latency is longer when counter-stereotype information is processed [40]. In the present study, we hypothesized that the LPP would reflect participants' evaluations of maternal stimuli. Specially, *mother* words would elicit a larger and later LPP in the *mother + bad* condition than in the *mother + good* condition due to the violation of the positive attitude towards the mother in the former condition. By contrast, people usually hold neutral attitudes towards non-specific others [42], [43]. Therefore, we expected that the LPP amplitude or its latency would not differ between the *others + good* and *others +bad* conditions.

In short, we investigated the neural correlates underlying maternal stimulus processing. We hypothesized that the attentional bias for maternal stimuli would be reflected in the P200 and N200 components and the positive evaluation bias for maternal stimuli would be reflected in the LPP.

## Method

### Ethics statement

The experimental protocol was approved by the Institutional Review Board (IRB) at the Institute of Psychology, Chinese Academy of Sciences. We explained the experimental procedure to each participant after he or she arrived at the lab. Moreover, informed written consent was obtained from each participant before the experiment.

### Participants

Twenty-seven college students (19–25 years old, 12 males, all right-handed) participated in this study as paid volunteers. None had a history of neurological or psychiatric disorders. All had normal or corrected-to-normal vision. Data from 4 participants (3 males) were not included in final analysis because of technical problems during data acquisition. As a result, the final sample consisted of twenty-three participants (9 males; age, *M* = 21.8 years, *SD*  = 1.7 years).

### Materials

We selected 170 Chinese words as stimuli: 5 *mother* words (ma, mother, mama, ama (“阿妈”, means mom) and niang (“娘”, means mom), 5 *others* words (he, him, his, other (“他人”, means other people) and other (“别人”, means other people), 80 *good* or *positive* and 80 *bad* or *negative* attributes. Most attributes were selected from the Chinese version of personal trait words provided by Anderson [44]. The remaining attributes were selected from a Chinese attribute list developed for a prior study examining implicit and explicit self-enhancement [45].

The familiarity of the target category was manipulated from three perspectives. First, all the characters we used are frequently used in daily life (i.e., they were among the top 8% of the 10,241 characters in the “Combined character frequency list of Classical and Modern Chinese, see the link http://lingua.mtsu.edu/chinese-computing/statistics/char/list.php?Which=TO”). Second, the frequency of *mother* words is not significantly different from that of *others* words (8,001 vs. 321,369, *t*_(4.002)_ = 2.44, *p* = .071) in the Chinese language, according to the word frequency Dictionary of Chinese characters and words (developed by the International R&D Center for Chinese Education, http://nlp.blcu.edu.cn). Third, to make sure that participants were familiar with both the *mother* and *others* words before the formal experiment, we required them to achieve response accuracy higher than 85% in the practice task.

### Procedure

Two GNATs consisted of four blocks: *mother + good*, *mother + bad*, *others + good* and *others + bad*, measuring processing of *mother* (*mother + good* and *mother + bad*) and *others* (*others + good* and *others + bad*), respectively. In each block, four identical types of stimuli were presented randomly on the computer screen one by one. Different blocks, however, required participants to respond to different pairs of stimuli (targets) but ignore other stimuli (distracters). For instance, in the *mother + good* block, participants needed to press the space bar if a stimulus was a *mother* word or a *good* word (e.g., *mother* or *delight*), but did nothing if a stimulus was an *others* word or a *bad* word (e.g., *he* or *bragging*). The order of the blocks was counterbalanced across participants. Before each experimental block, participants worked through pilot trials to become familiar with the task.

Each block included 320 trials. The stimulus presented in each trial was selected from four types of concepts with equal probability. Each of the five target category words (*mother* and *others*) was presented 16 times. The attribute words (*good* and *bad*) were presented without repetition. Therefore, there were 160 trials that presented target category words and the other 160 trials presented attributed words. The ratio of signal to noise was 1∶1 in each block.

**Figure 1:**
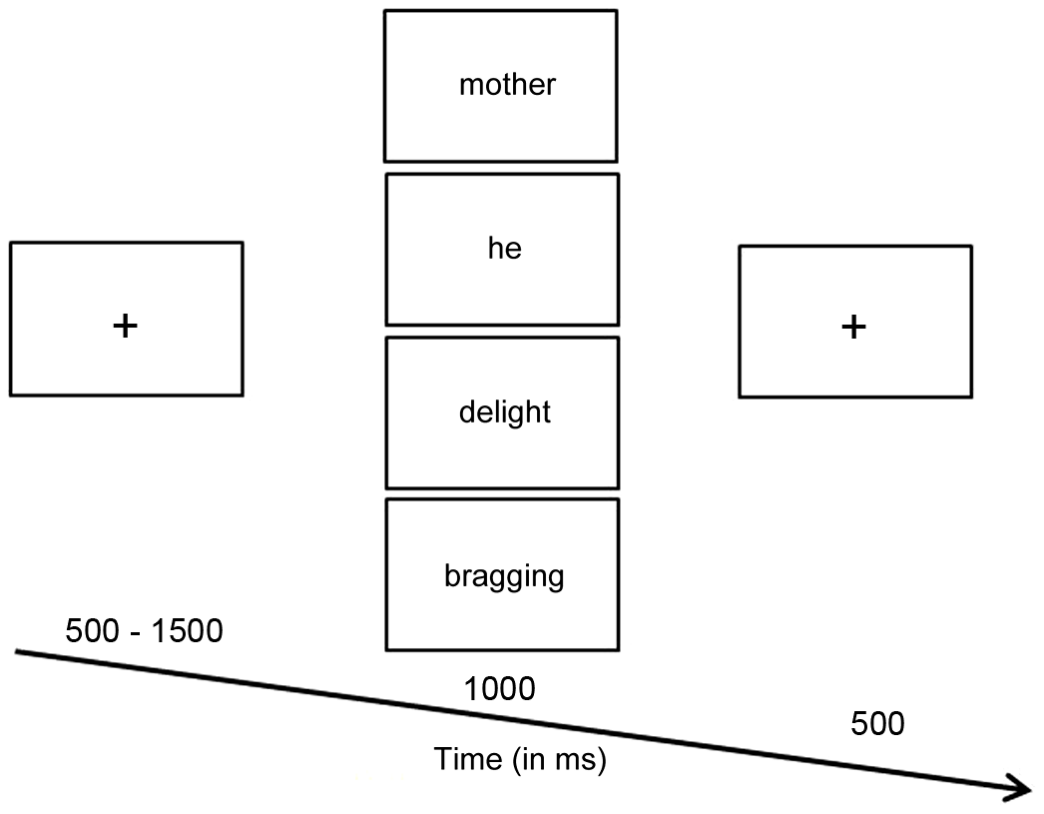
Illustration of the experimental procedure.

Figure 1 shows an example of the trial stimulus presentations. Each trial started with a fixation (a cross “+”) on the center of the screen appearing for a random duration between 500 and 1500 ms. After that, the stimulus word was presented on the center of the screen for 1000 ms, and the participants were required to press the SPACE bar if the stimulus word was a target item. Finally, the second fixation was presented for 500 ms. Thereafter, a new trial then started with a fixation.

### EEG Data Recording and Analysis

The continuous electroencephalogram (EEG) was recorded from 64 scalp sites using Ag/AgCl electrodes mounted in an elastic cap (NeuroScan Inc.), with an online reference to the right mastoid and off-line algebraic re-reference to the average of left and right mastoids. The vertical electrooculogram (VEOG) and horizontal electrooculogram (HEOG) were recorded from two pairs of electrodes, with one placed above and below the left eye, and another 10 mm from the outer canthi of each eye. All interelectrode impedances were maintained below 5 kΩ. The EEG and EOG were amplified using a 0.05–100 Hz bandpass and continuously sampled at 500 Hz/channel.

During the off-line analysis, the EEG data were digitally filtered with a 30 Hz low-pass filter. Ocular artifacts were removed from the filtered EEG data using a regression procedure implemented in the Neuroscan software [46]. The onsets of the stimuli were set as the zero points, and the continuous EEG data were epoched into periods of 1000 ms including a 200-ms pre-stimulus baseline. Trials with artifacts due to eye blinks, amplifier clipping, and electromyographic (EMG) activity exceeding ±100 µV were excluded from averaging. In addition, trials where a participant had responded incorrectly were excluded from the final averaging. The mean percentage of trials excluded from averaging across the four blocks was less than 2% (*M* = 1.8%, *SD*  = 2.1%). After that, the ERPs for category words (*mother* or *others*) with a Go response from the four blocks were averaged separately. Finally, two types of ERPs for each of the category words were obtained.

To examine attentional processing of maternal information, two ERP components, the P200 (within 140–210 ms) and N200 (within 250–350 ms), were examined. Previous studies showed that the P200 is generally found to be maximal around the frontal region of the scalp (e.g., [26], [27]). Empirical evidence suggests that the anterior N200 may reflect attentional allocation processes, including the attentional selection of salient physical properties of items made by task manipulations [47], [48] and the properties of social stimuli that are of inherent motivation/salience, such as out-group membership information (for a review, see [49]). Therefore, the peak amplitudes of P200 and N200 from 9 anterior sites (F3, FZ, F4, FC3, FCZ, FC4, C3, CZ and C4) and analyzed, respectively. These amplitudes from two components were entered into a four-way ANOVA (target category (*mother* vs. *others*) × attribute (*good* vs. *bad*) × Anterior-Posterior (F vs. FC vs. C) × Laterality (left vs. midline vs. right)), respectively.

To examine the evaluative processing of maternal stimuli, the peak latency and mean amplitude of LPP was measured. As this component is generally found to be maximal around the posterior area of the scalp (for reviews, see [32]–[35]), we selected 12 central parietal sites (C3, CZ, C4, CP3, CPZ, CP4, P3, PZ, P4, PO3, POZ and PO4) to measure its mean amplitude from 400 ms to 600 ms. These peak latencies and mean amplitudes were entered into a four-way ANOVA (target category (*mother* vs. *others*) × attribute (*good* vs. *bad*) × Anterior-Posterior (C vs. CP vs. P vs. PO) × Laterality (left vs. midline vs. right)), respectively.

Additionally, to check the physical properties differences between maternal stimuli and *others* stimuli, P100 and anterior N100 were assessed and analyzed. Peak amplitude of P100 (within 80–120 ms) was measured from 3 occipital sites (O1, OZ and O2) and then entered into a three-way ANOVA (target category (*mother* vs. *others*) × attribute (*good* vs. *bad*) × Laterality (left vs. midline vs. right)). Meanwhile, peak amplitude of N100 (within 90–130 ms) were measured from 9 anterior sites (F3, FZ, F4, FC3, FCZ, FC4, C3, CZ and C4) and then submitted into a four-way ANOVA (target category (*mother* vs. *others*) × attribute (*good* vs. *bad*) × Anterior-Posterior (F vs. FC vs. C) × Laterality (left vs. midline vs. right)).

For all the analyses listed below, the significance level was set at 0.05. Greenhouse–Geisser correction was used to compensate for sphericity violations when appropriate. Post-hoc analyses were conducted to explore the interaction effects. Partial eta-squared (*η^2^*) was reported to demonstrate the effect sizes of significant results in the ANOVA tests.

## Results

### Behavioral Results

To examine whether attentional and evaluative factors contribute to the behavioral response, we performed an ANOVA on accuracy and reaction time to *mother* and *others* words in Go trials with target category (*mother* vs. *others*) and attribute (*good* vs. *bad*) as two within-subject factors, respectively. Regarding accuracy, participants performed at 96.63% accuracy in four conditions, and no significant difference was found across conditions, all *F*s <0.11 and all *p*s>.35. Regarding reaction time, the category effect was not significant, *F*_(1, 22)_ = 2.31, *p* = .143, *partial η^2^* = 0.095, but the attribute effect was significant, *F*_(1, 22)_ = 5.48, *p* = .029, *partial η^2^* = 0.20. Targets paired with *good* words elicited faster responses (*M* = 474 ms, *SD*  = 44) than targets paired with *bad* words (*M* = 490 ms, *SD*  = 52). More importantly, the interaction between category and attribute was highly significant, *F*_ (1, 22)_ = 12.76, *p* = .002, *partial η^2^* = 0.37. Participants responded faster to *mother* words in the *mother + good* condition (*M* = 461 ms, *SD*  = 41) than in *mother + bad* condition (*M* = 492 ms, *SD*  = 50), *t*_(22)_ = −4.13, *p*<.001, but the reaction times for *others* words, between *others + good* (*M* = 488 ms, *SD*  = 47) and *others + bad* (*M* = 487 ms, *SD*  = 54), were not significantly different, *t*_(22)_ = .07, *p* = .945. These findings suggested that participants had positive attitudes to mother, which is consistent with previous findings [19]–[21].

### ERP Results

#### P100 and N100 amplitude

No significant difference was detected in peak amplitudes of the P100 (peaks at 98.33 ms) and anterior N100 (peaks at 98.50 ms), all *F*s<3.90, and all *p*s>.05. Therefore, we concluded that *mother* and *others* words were processed similarly in terms of physical properties, thus not affecting early visual processing.

#### P200 amplitude

**Figure 2:**
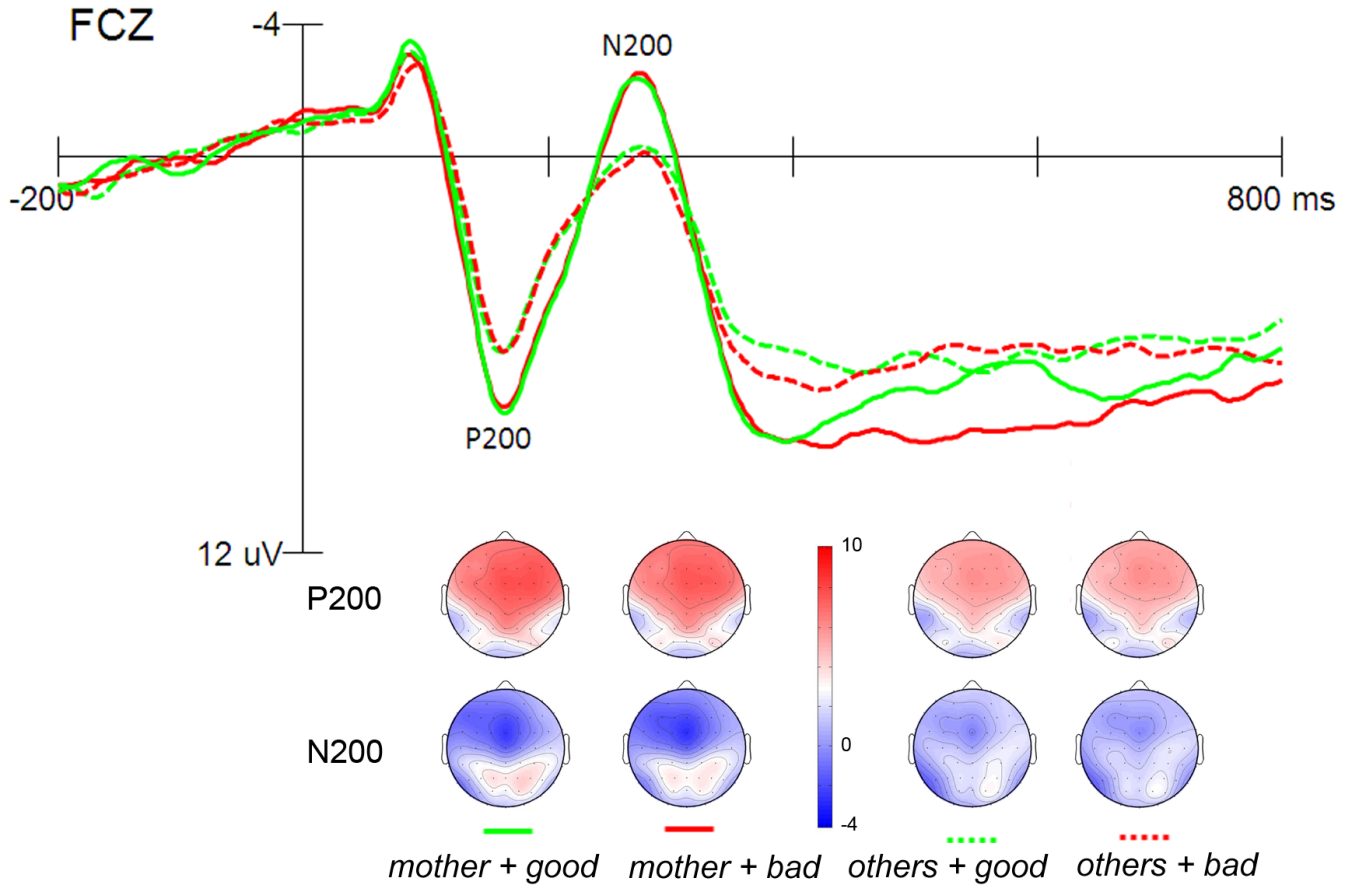
Grand averaged ERPs for target category words. The scalp topographies at peak latency for P200 and N200 of each condition are presented beneath.

The peak amplitudes of the P200 (peaks at 165.62 ms) within 140–210 ms were entered into a four-way ANOVA. Main effect of target category was significant, *F*_(1, 22)_ = 30.46, *p*<.001, *partial η^2^* = 0.58, with *mother* words eliciting larger P200 (*M* = 8.14 µV, *SD*  = 4.45) than *others* words (*M* = 6.34 µV, *SD*  = 3.72) (see Figure 2). Neither the main effect of attribute nor the interaction effect was significant, *F*_(1, 22)_ = 1.06, 0.61, *p* = .315,.442, *partial η^2^* = 0.05, 0.03, respectively.

#### N200 amplitude

The peak amplitudes of the anterior N200 (peaks at 279.67 ms) within 250–350 ms were entered into a four-way ANOVA. Main effect of target category was significant, *F*_(1, 22)_ = 14.86, *p* = .001, *partial η^2^* = 0.40, with *mother* words eliciting larger N200 (*M* = −1.92 µV, *SD*  = 4.80) than *other* words (*M* = −0.53 µV, *SD*  = 4.71) (see Figure 2). Neither the main effect of attribute nor the interaction effect was significant, *F*_(1, 22)_ = 0.01, 0.52, *p* = .932,.481, *partial η^2^* = 0.001,.023, for valence and interaction, respectively.

#### LPP amplitude

**Figure 3:**
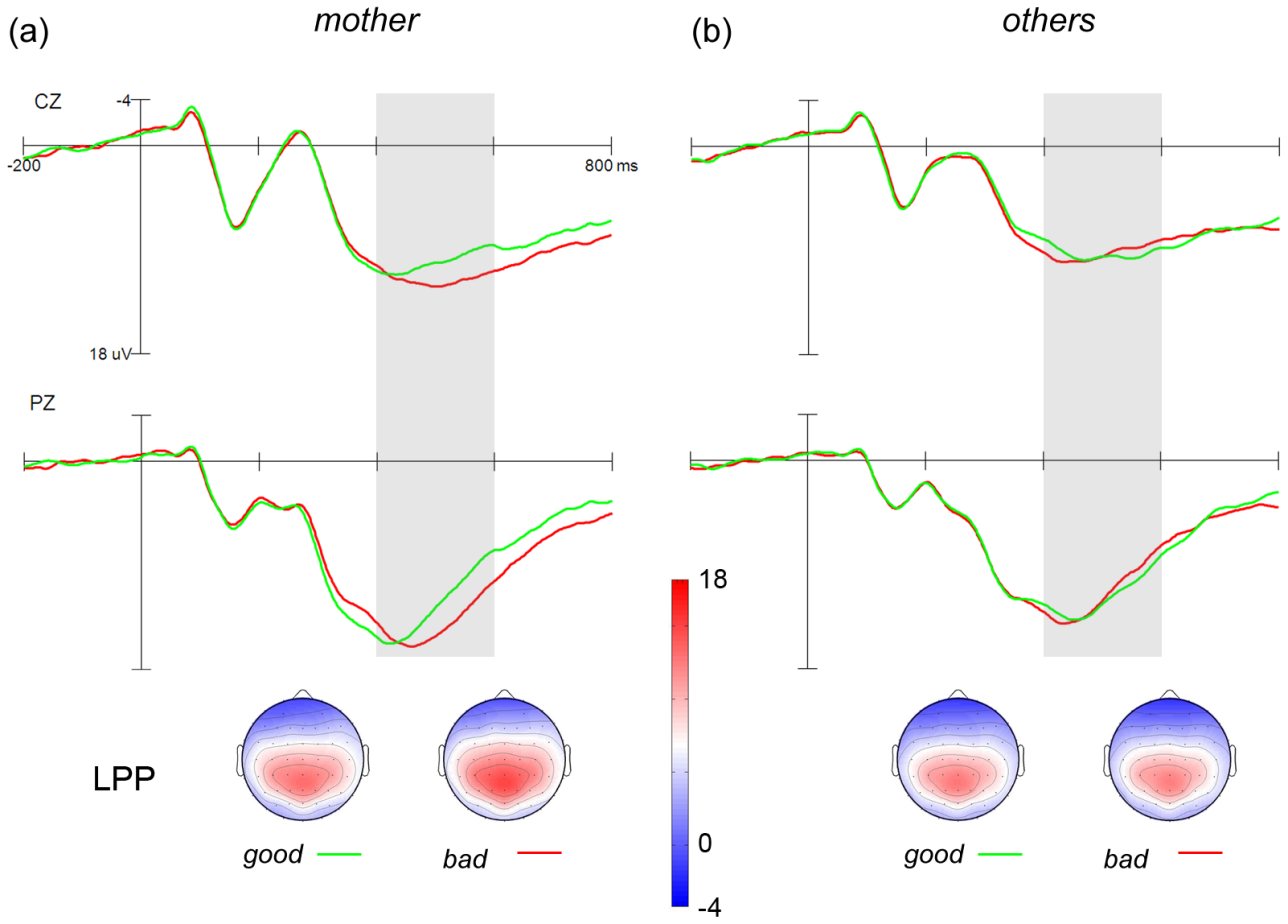
Grand averaged ERPs for target category words. The light gray shaded areas indicate the time window for the detection of the LPP component. The scalp topographies of each condition are presented beneath.

The grand averaged ERPs to *mother* and *others* words from Go trials are shown in Figure 3a and Figure 3b, respectively. Mean amplitude of LPP was measured and submitted to a four-way ANOVA. The main effect of target category was significant, *F*_(1, 22)_ = 20.41, *p*<.001, *partial η^2^* = 0.48, with *mother* words eliciting larger LPP than *others* words (*mother*: *M* = 10.24 µV vs. *others*: *M* = 8.90 µV). The main effect of attribute was not significant, *F*_(1, 22)_ = 1.79, *p* = .195, *partial η^2^* = 0.08. The interaction effect for category and attribute was significant, *F*_(1, 22)_ = 8.86, *p* = .007, *partial η^2^* = 0.29. Further analyses showed that *mother* in the *mother + bad* condition (*M* = 10.90 µV) elicited larger LPP than in the *mother + good* condition (*M* = 9.59 µV), *F*_(1, 22)_ = 7.21, *p* = .014, *partial η^2^* = 0.25. By contrast, no significant difference was found in LPP mean amplitude between the *others + bad* (*M* = 8.75 µV) and *others + good* conditions (*M* = 9.06 µV), *F*_(1, 22)_ = 0.49, *p* = .493, *partial η^2^* = 0.02.

#### LPP latency

Peak latencies of LPP were measured and submitted to a four-way ANOVA. The main effect of target category was not significant, *F*_(1, 22)_ = 2.73, *p* = .113, *partial η^2^* = .11. The main effect of attribute was significant, *F*_(1, 22)_ = 6.22, *p* = .021, *partial η^2^* = .22, with target category words paired with *good* words eliciting an earlier LPP (*M* = 428 ms) than those paired with *bad* words (*M* = 441 ms). The category by valence interaction was significant, *F*_(1, 22)_ = 13.15, *p* = .001, *partial η^2^* = 0.37. Further analyses showed that *mother* in the *mother + bad* condition (*M* = 447 ms) elicited a later LPP than in the *mother + good* condition (*M* = 414 ms), *F*_(1, 22)_ = 26.26, *p*<.001, *partial η^2^* = .54. By contrast, no significant difference was found in LPP latency between the *others + bad* (*M*  =  435 ms) and *others + good* conditions (*M* = 442 ms), *F*_(1, 22)_ = 0.54, *p* = .471, *partial η^2^* = 0.024.

### Results of the two gender neutral "others" items

One potential concern with the current results was the “woman are wonderful” effect, in which both males and females have a general tendency to associate women with more positive attributes and expectations than men [50]. It is possible that the *mother*/*others* manipulation was confounded with female/male in our findings, seeing that three of the *others* stimuli were masculine (he, him, his). One way to rule out this possibility was to directly compare the *mother* stimuli with the two non-masculine *others* stimuli. Specifically, we selected the trials elicited by the two gender-neutral words (“他人” and “别人”) under *others + bad* and *others + good* conditions, respectively. Then, we compared the reaction time and the ERP components elicited by *mother* words with those elicited by these two *others* words, respectively. The results were similar to those reported above. Specifically, participants showed positive bias to *mother* at behavioral level. ERP results showed that they allocated more attentional resources to maternal stimuli, and they held positive evaluations of *mother*. The detailed results are listed below.

### Behavioral result

The result showed that participants made faster responses to *mother* words (*M* = 476 ms, *SD*  = 42) than to *others* words (*M* = 500 ms, *SD*  = 50), *F*_(1, 22)_ = 8.04, *p* = .01, *partial η^2^* = 0.27. More importantly, the category × attribute interaction effect was significant, *F*_ (1, 22)_ = 11.13, *p* = .003, partial *η^2^* = 0.34. Further tests showed that participants responded faster to *mother* words in the *mother + good* condition (*M* = 461 ms, *SD*  = 41) than in the *mother + bad* condition (*M* = 492 ms, *SD*  = 50), *t*_(22)_ = −4.13, *p*<.001; whereas the reaction times for *others* words, between *others + good* (*M* = 501 ms, *SD*  = 47) and *others + bad* (*M* = 499 ms, *SD*  = 63), were not significantly different, *t*_(22)_ = .168, *p* = .868.

### ERP results

#### P200

The peak amplitudes of the P200 within 140–210 ms were entered into a four-way ANOVA. The main effect of category was significant, *F*_(1, 22)_ = 20.23, *p*<.001, *partial η^2^* = 0.479, with *mother* words eliciting larger P200 than *others* words (*mother*: *M* = 8.14 µV vs. *others*: *M* = 6.70 µV). Neither the main effect of attribute nor the interaction effect were significant, *F*_(1, 22)_ = 0.465, 0.716, *p* = .503,.406, *partial η^2^* = 0.021, 0.032, respectively.

#### N200

The peak amplitudes of the anterior N200 within 250–350 ms were entered into a four-way ANOVA. Main effect of target category, main effect of attribute, and the Category × Attribute interaction were not significant, all *F*s<1.8, all *p*s>.2. However, a significant Category × Laterality interaction effect was found, *F*_(2, 44)_ = 8.88, *p* = .001, *partial η^2^* = .29. Further analysis showed that the category effect was only significant over left area, *F*_(1, 22)_ = 5.63, *p* = .027, *partial η^2^* = .204, with *mother* words eliciting larger N200 than *others* words in this area.

#### LPP

Mean amplitude of LPP was measured and submitted into a four-way ANOVA. The main effect of target category was significant, *F*_(1, 22)_ = 19.41, *p*<.001, *partial η^2^* = 0.47, with *mother* words elicited larger LPP than *others* words (*mother*: *M* = 10.24 µV vs. *others*: *M* = 8.47 µV). The main effect of attribute was not significant, *F*_(1, 22)_ = 1.40, *p* = .25, *partial η^2^* = 0.06. The category by attribute interaction was significant, *F*_(1, 22)_ = 5.92, *p* = .024, *partial η^2^* = 0.21. Further analyses showed that *mother* in the *mother + bad* condition elicited a larger LPP (*M* = 10.90 µV) than in the *mother + good* condition (*M* = 9.59 µV), *F*_(1, 22)_ = 7.21, *p* = .014, *partial η^2^* = 0.25. By contrast, no significant difference was found in LPP mean amplitudes between the *others + bad* (*M* = 8.34 µV) and *others + good* conditions (*M* = 8.61 µV), *F*_(1, 22)_ = 0.20, *p* = .66, *partial η^2^* = 0.009.

In addition to the *mother*/*others* manipulation, since the majority of the sample were female and the “women are wonderful” effect is stronger in females than males [51], the gender ratio (female: male  = 14∶9) might have confounded the result. To rule it out, we averaged the data of female and male participants separately and then re-analyzed the original behavioral and ERPs result with gender (female vs. male) as a group factor. If the findings indeed reflected the maternal/others effect, then there should be no significant gender difference. Otherwise, if the findings were cofounded with the “women are wonderful” phenomenon, a gender effect should manifest on behavioral and/or neural response. The result showed that the gender effect was not significant after being entered into ANOVA tests. Moreover, adding this factor to analyses does not affect our major findings of either the behavioral or the ERPs results (including the P200, N200, and LPP components), which suggested that the gender factor did not affect the behavioral and neural response.

## Discussion

The present study aimed to investigate attentional and evaluative processing of maternal stimuli. We examined whether there was an attentional preference to maternal information after controlling for familiarity, and we sought to reduce social desirability in the evaluations of maternal stimuli by using an implicit evaluative task and measuring electrophysiological responses. The behavioral data in the formal experiment, including accuracy and response time, supported the experimental control of stimulus familiarity. Target category effects were not significant for either measure, which would be difficult to explain if *mother* words were more familiar than the *others* words. Thus, the neural response to *mother* words likely reflects the specificity of maternal stimulus processing instead of stimulus familiarity.

The behavioral results also suggested that participants indeed had a positive attitude towards maternal stimuli, which was consistent with previous findings [19]–[21]. That is, participants responded faster when *mother* words were grouped with *good* attributes than when they were grouped with *bad* attributes; whereas no significant difference was found between *others* paired with *good* or *bad* attributes.

The ERP results revealed two major findings. First, *mother* words garnered more attention than non-specific *others* words in both the early and late stages of processing. The P200/N200 and the LPP, respectively, were larger in amplitude for *mother* than for *others* words. Second, the positive attitude towards maternal stimuli emerged in the evaluative stage. The LPP amplitude was larger for the *mother + bad* than *mother + good* condition.

Previous research has consistently demonstrated that maternal stimuli, such as participants' mothers' faces, receive more attentional resources and deeper processing compared with *others* stimuli, as shown by indexes such as looking time [17], [18] and blood-oxygen-level-dependent (BOLD) signals [16]. Such preferences were usually attributed to familiarity. However, the present study extends this literature by showing that this kind of phenomenon occurs even after controlling for the level of familiarity. We believe that the selectively deep processing of maternal information was due to the critical role of mothers in human life and the relationships between the participants and their respective mothers.

The current study also revealed the temporal course for this attentional bias using ERPs. Compared with *others*, *mother* elicited larger P200 and N200 components, suggesting that maternal information received considerable attention at an early stage of stimulus processing. Additionally, the augmented LPP amplitude for *mother* compared with *others* also reflected greater attentional resources allocation to *mother* during later cognitive processing stages. Because the five items of maternal stimuli were specific and concrete, whereas the *others* items are non-specific and abstract, it is possible that the attentional preference to maternal stimuli resulted from the concreteness of the words. However, we do not think so. Several empirical studies have shown that concrete words differ from abstract words at a late processing stage, with concrete words eliciting a larger N400 (between 300 and 550 ms) and a larger N700 (between 550 and 800 ms) than abstract words post stimulus onset, but not at the attentional stage (e.g., refs [52], [53]). Therefore, the LPP difference to maternal stimuli indexes attention allocation to *mother* and does not index the differences in word concreteness. Additionally, the attentional bias we observed is consistent with the results of previous studies where stimulus salience was operationalized as a function of the degree of self-relevance [31], [54]–[58].

In the present study, the evaluative processing of mother was operationalized by the association between *mother* words and evaluative attributes. Behavioral results from this study and other studies (e.g., refs [19]–[21]) suggest that people evaluate mother positively. In the *mother + bad* condition, *mother* words were assigned to negative attributes, which was incompatible with the intrinsic positive attitude towards the mother [19]–[21]. This evaluative incompatibility in the present study resulted in an augmented LPP. This finding was in line with earlier findings that the LPP reflects people's evaluations of social targets, such as people of different races or genders [40], [41], [59]. Additionally, the LPP latency was longer when incompatible evaluative information (*mother + bad*) is processed compared with the compatible evaluative information (*mother + good*), which is also in line with previous findings [41]. Our study shows that the evaluative processing of maternal stimuli occurs during a later stage of processing. For *others* stimuli, however, the LPP amplitudes and latency were not significantly different between the *others + bad* and *others + good* conditions, which was consistent with earlier claims that people hold a neutral attitude towards non-specific others [42], [43].

Given that the RTs in the *mother + bad* condition were significantly longer than those in the *mother + good* condition, two other potential factors might account for the ERP differences between these two conditions: overt motor response speed and task difficulty. We have two reasons for disagreeing with the former issue. First, participants carried out the same key-pressing responses to identical stimuli (*mother* words) in both the *mother + bad* and *mother + good* conditions. Second, if the LPP amplitude difference was caused merely by the speed difference of the same motor response, there would be no significant difference in the amplitude of the ERP wave before a response between these two conditions. To directly test this hypothesis, we set the response point as the time zero and then measured and analyzed the amplitude of the ERP wave before the motor response over the centro-parietal area. The results revealed that the ERP wave in the *mother + bad* condition was larger than that in the *mother + good* condition, and no significant difference between the *others + bad* and *others + good* conditions. These results suggest that the differences in LPP amplitudes did not result from differences in overt motor response speed (please refer to Text S1 and Figure S1 for details).

Concerning the issue of task difficulty, the behavioral and neural findings between *mother + bad* and *mother + good* conditions might be due to differences in task difficulty. However, the source of this difficulty is worth noting. In the field of experimental psychology, task difficulty has been manipulated in several ways, including the stimulus characteristics [60], [61], the physical effort required in the task [62], the understandability of the stimulus content [63], the length of time allotted to make a response [64], [65], the number of cognitive operations needed to complete the task [66], the complexity of mental operations (e.g., [67]), and the strength of the inconsistency between different response tendencies [68], [69]. In the current study, the differences in difficulty levels were not caused by the stimulus characteristics or the mental steps necessary for response implementation, but they varied as a function of the degree of mental inconsistency. That is, asking participants to respond to *mother* and *bad* words, the *mother* + *bad* condition creates a scenario in which automatic evaluation (i.e., associating maternal stimuli with positive descriptions) strongly conflicts with task-demand evaluation. This may have increased the task difficulty, thus leading to longer response times and larger LPP amplitudes compared to the *mother* + *good* condition, with no significant differences between *others* + *good* and *others* + *bad* conditions. Thus, even though our major findings might be interpreted in terms of task difficulty, we argue that these differences in difficulty levels between the *mother* conditions were the result of the internal positive attitude towards maternal stimuli.

Although the current study aimed to examine the maternal/other effect, it is possible that the “women are wonderful” effect [51], rather than the maternal/other effect, might explain our results. First, three of the five “*others*” items were masculine. Second, because the sample of the present study largely consisted of female participants and the “women are wonderful” effect is stronger in females than males [50], the gender ratio (female: male  = 14∶9) of the sample might have affected the result. However, we re-analyzed the data in order to rule out the potential effect of masculine words and the gender ratio of the sample. The first analysis showed that when we selectively made comparison between *mother* words and two gender-neutral *others* words, the results were essentially the same. Although the effect of category on the N200 amplitude was only significant over left hemisphere, we suggest this asymmetry may disappear in a larger sample. Alternatively, the attention to maternal information might depend more on left hemisphere, but we believe that this is unlikely because the contrast of N200 between *mother* words and all five *others* words did not show a left asymmetry. Regarding the low spatial resolution of ERP technique, future brain-imaging studies would be more appropriate for clarifying this issue. With respect to the gender ratio, a second analysis showed that the gender effect was not significant, i.e., it did not moderate the behavioral or the ERPs results. Thus, the results from these analyses both did not challenge our initial findings and interpretations.

The results suggest that both attentional and evaluative components are involved in processing maternal stimuli. The attentional distinction of *mother* from non-specific *others* occurs immediately after stimulus presentation, which is in line with the proposal that people spontaneously group their perceptions of others by relationship status [70]. The evaluative components contributed to the later processing of the maternal stimuli. The initial recognition and later evaluation of maternal stimuli probably jointly contribute to constructions of a complex representation of the mother, which in turn affects participants' cognition, emotion, motivation, and behaviors.

One limitation of the present study was that we only compared *mother* and *others* stimuli. We do not know whether such responses are specific to the mother or could be generalized to significant others, such as the father. The father's role as attachment figure has been emphasized recently (for a review, see [71]). It is likely that the processing of paternal stimuli involves cognitive and affective components similar to the processing of maternal stimuli. Future studies could extend our findings by adding another target, such as *father*, to the task design.

Another limitation was that we only tested adults. Thus, we could not test whether the current findings and conclusions can be generalized across different ages. Given that for most children, mothers are the primary attachment figures, whereas for adults the mother is generally ranked lower than romantic partners [9], it is possible that the processing of maternal stimuli is different between children and adults. However, this may not be the case, since there is some evidence that the neural activation evoked by maternal information is not modulated by age [72]. Thus, the response to the mother observed in the present study may also be generalizable to younger people. Future studies that examine the behavioral and neural response to maternal stimuli in children could help clarifying this issue.

In conclusion, our results indicate that maternal stimuli affect people's attentional and evaluative processing in a short time interval (less than one second). Such attentional preference and evaluative processing were not a result of stimulus familiarity or social desirability bias, but they were driven by the critical role of mother in human life. Moreover, we found that instead of using stimuli directly related to participants' own mother, such as the mother's face, just presenting symbolic stimuli that describe the mother elicits biased processing. This suggests that a more abstract, general, and typical mother concept—not just concrete and specific maternal information—can receive instant processing.

## Supporting Information

Text S1 **Response-locked ERP analysis for** ***mother*** **and** ***others*** **words with Go response.**
(DOCX)

Figure S1 **Grand averaged ERPs for target category words.** The light gray shaded areas indicate the time window for the detection of the LPP component.
(TIF)

## References

[1] Murray L, Fiori-Cowley A, Hooper R, Cooper P (1996) The Impact of Postnatal Depression and Associated Adversity on Early Mother-Infant Interactions and Later Infant Outcome. Child Dev 67: 2512–2526. 9022253

[2] Oweis A (2008) Maternal Depression and Infant Temperament Characteristics. MCN Am J Matern Child Nurs 33: 393–393.

[3] Rautava S, Kalliomäki M, Isolauri E (2002) Probiotics During Pregnancy and Breast-feeding Might Confer Immunomodulatory Protection Against Atopic Disease in the Infant. J Allergy Clin Immunol 109: 119–121. 11799376 10.1067/mai.2002.120273

[4] Yeung LTF, King SM, Roberts EA (2001) Mother-to-infant Transmission of Hepatitis C Virus. Hepatology 34: 223–229. 11481604 10.1053/jhep.2001.25885

[5] Hofer MA (1994) Early Relationships as Regulators of Infant Physiology and Behavior. Acta Paediatr 83: 9–18. 10.1111/j.1651-2227.1994.tb13260.x 7981480

[6] Bowlby J (1982) Attachment and loss. Vol. 1: Attachment (2nd Ed.). New York: Basic Books (new printing, 1999, with a foreword by Allan N. Schore; originally published in 1969).

[7] Steeger CM, Gondoli DM (2013) Mother-Adolescent Conflict as a Mediator Between Adolescent Problem Behaviors and Maternal Psychological Control. Dev Psychol 49: 804–814. 22612432 10.1037/a0028599 PMC4203150

[8] Yap MB, Allen NB, O'Shea M, di Parsia P, Simmons JG, et al (2011) Early Adolescents' Temperament, Emotion Regulation During Mother-child Interactions, and Depressive Symptomatology. Dev Psychopathol 23: 267–82. 21262053 10.1017/S0954579410000787

[9] Doherty NA, Feeney JA (2004) The Composition of Attachment Networks Throughout the Adult Years. Pers Relatsh 11: 469–488.

[10] Antonucci TC, Akiyama H, Takahashi K (2004) Attachment and Close Relationships Across the Life Span. Attach Hum Dev 6: 353–370. 15764124 10.1080/1461673042000303136

[11] Mallers MH, Charles ST, Neupert SD, Almeida DM (2010) Perceptions of Childhood Relationships with Mother and Father: Daily Emotional and Stressor Experiences in Adulthood. Dev Psychol 46: 1651–61. 20873925 10.1037/a0021020 PMC3468907

[12] Field TM, Cohen D, Garcia R, Greenberg R (1984) Mother-stranger Face Discrimination by the Newborn. Infant Behav Dev 7: 19–25.

[13] Bushneil IWR, Sai F, Mullin JT (1989) Neonatal Recognition of the Mother's Face. Br J Dev Psychol 7: 3–15.

[14] Walton GE, Bower NJA, Bower TGR (1992) Recognition of Familiar Faces by Newborns. Infant Behav Dev 15: 265–269.

[15] de Haan M, Nelson CA (1997) Recognition of the Mother's Face by Six-month-old Infants: a neurobehavioral study. Child Dev 68: 187–210. 9179998

[16] Arsalidou M, Barbeau EJ, Bayless SJ, Taylor MJ (2010) Brain Responses Differ to Faces of Mothers and Fathers. Brain Cogn 74: 47–51. 20621407 10.1016/j.bandc.2010.06.003

[17] Bushnell IWR (2001) Mother's Face Recognition in Newborn Infants: Learning and Memory. Infant Behav Dev 10: 67–74.

[18] Pascalis O, de Schonen S, Morton J, Deruelle C, Fabre-Grenet M (1995) Mother's Face Recognition by Neonates: A Replication and an Extension. Infant Behav Dev 18: 79–85.

[19] Dehart T, Pelham B, Fiedorowicz L, Carvallo M, Gabriel S (2011) Including Others in the Implicit Self: Implicit Evaluation of Significant Others. Self Identity 10: 127–135.

[20] Zhou AB, Wu HF, Shi Z, Li Q, Liu PR, et al (2010) The Contrastive Study of Mother-reference Effect in the Depth of Processing and Incidental Encoding Conditions. Psychological Exploration 03: 39–44 (in Chinese)..

[21] Tottenham N, Shapiro M, Telzer EH, Humphreys KL (2012) Amygdala Response to Mother. Dev Sci 15: 307–319. 22490172 10.1111/j.1467-7687.2011.01128.x PMC3522470

[22] Luck SJ (2005) An Introduction to the Event-related Potential Technique. Cognitive neuroscience. Cambridge, Mass.: MIT Press. xii, 374 p.

[23] Nosek BA, Banaji MR (2001) The Go/No-go Association Task. Soc Cogn 19: 625–666.

[24] Banfield JF, van der Lugt AH, Munte TF (2006) Juicy Fruit and Creepy Crawlies: An Electrophysiological Study of the Implicit Go/NoGo Association Task. Neuroimage 31: 1841–1849. 16581266 10.1016/j.neuroimage.2006.02.017

[25] van der Lugt AH, Banfield JF, Osinsky R, Münte TF (2012) Brain Potentials Show Rapid Activation of Implicit Attitudes Towards Young and Old People. Brain Res 1429: 98–105. 22088825 10.1016/j.brainres.2011.10.032

[26] Hu X, Wu H, Fu G (2011) Temporal Course of Executive Control When Lying about Self- and Other-referential Information: An ERP Study. Brain Res 1369: 149–57. 21059343 10.1016/j.brainres.2010.10.106

[27] Meixner JB, Rosenfeld JP (2010) Countermeasure Mechanisms in a P300-based Concealed Information Test. Psychophysiology 47: 57–65. 19761523 10.1111/j.1469-8986.2009.00883.x

[28] Chen AT, Weng XC, Yuan JJ, Lei X, Qiu J, et al (2008) The Temporal Features of Self-referential Processing Evoked by Chinese Handwriting. J Cogn Neurosci 20: 816–827. 18201135 10.1162/jocn.2008.20505

[29] Keyes H, Brady N, Reilly RB, Foxe JJ (2010) My Face or Yours? Event-related Potential Correlates of Self-face Processing. Brain Cogn 72: 244–254. 19854553 10.1016/j.bandc.2009.09.006

[30] Chen J, Yuan J, Feng T, Chen A, Gu B, et al (2011) Temporal Features of the Degree Effect in Self-relevance: Neural Correlates. Biol Psychol 87: 290–295. 21470572 10.1016/j.biopsycho.2011.03.012

[31] Chen J, Zhang Y, Zhong J, Hu L, Li H (2013) The Primacy of the Individual Versus the Collective Self: Evidence from an Event-related Potential Study. Neurosci Lett 535: 30–34. 23313591 10.1016/j.neulet.2012.11.061

[32] Kok A (2001) On the Utility of P3 Amplitude as a Measure of Processing Capacity. Psychophysiology 38: 557–577. 11352145 10.1017/s0048577201990559

[33] Polich J (2007) Updating P300: An Integrative Theory of P3a and P3b. Clin Neurophysiol 118: 2128–48. 17573239 10.1016/j.clinph.2007.04.019 PMC2715154

[34] Herrmann CS, Knight RT (2001) Mechanisms of Human Attention: Event-related Potentials and Oscillations. Neurosci Biobehav Rev 25: 465–476. 11595268 10.1016/s0149-7634(01)00027-6

[35] Verleger R, Jaśkowski P, Wascher E (2005) Evidence for an Integrative Role of P3b in Linking Reaction to Perception. J Psychophysiol 19: 165–181.

[36] Cacioppo JT, Crites SL, Berntson GG, G. H. Coles M (1993) If Attitudes Affect How Stimuli Are Processed, Should They Not Affect the Event-Related Brain Potential? Psychol Sci 4: 108–112.

[37] Cacioppo JT, Crites SL, Gardner WL, Berntson GG (1994) Bioelectrical Echoes from Evaluative Categorizations: I. A Late Positive Brain Potential that Varies as a Function of Trait Negativity and Extremity. J Pers Soc Psychol 67: 115–125. 8046583 10.1037//0022-3514.67.1.115

[38] Ito TA, Cacioppo JT (2000) Electrophysiological Evidence of Implicit and Explicit Categorization Processes. J Exp Soc Psychol 36: 660–676.

[39] Lust SA, Bartholow BD (2009) Self-reported and P3 Event-related Potential Evaluations of Condoms: Does What We Say Match How We Feel? Psychophysiology 46: 420–4. 19207201 10.1111/j.1469-8986.2008.00775.x PMC4692251

[40] Bartholow BD, Dickter CL, Sestir MA (2006) Stereotype Activation and Control of Race Bias: Cognitive Control of Inhibition and Its Impairment by Alcohol. J Pers Soc Psychol 90: 272–287. 16536651 10.1037/0022-3514.90.2.272

[41] Ito TA, Thompson E, Cacioppo JT (2004) Tracking the timecourse of social perception: the effects of racial cues on event-related brain potentials. Pers Soc Psychol Bull 30: 1267–1280.. 15466600 10.1177/0146167204264335

[42] Karpinski A (2004) Measuring Self-esteem Using the Implicit Association Test: The role of the other. Pers Soc Psychol Bull 30: 22–34. 15030640 10.1177/0146167203258835

[43] Pinter B, Greenwald AG (2005) Clarifying the Role of the "Other" Category in the Self-esteem IAT. Exp Psychol 52: 74–9. 15779533 10.1027/1618-3169.52.1.74

[44] Anderson NH (1968) Likableness Ratings of 555 Personality-trait Words. J Pers Soc Psychol 9: 272–9. 5666976 10.1037/h0025907

[45] Zhao C (2008). Wai Xian Zi Wo Zeng Qiang Yu Nei Yin Zi Wo Zeng Qiang De Shen Jing Ji Zhi Yan Jiu [Neural Research of Explicit Self-esteem and Implicit Self-esteem]. Unpublished mater's thesis, Capital Normal University, Beijing, China.

[46] Semlitsch HV, Anderer P, Schuster P, Presslich O (1986) A Solution for Reliable and Valid Reduction of Ocular Artifacts, Applied to the P300 ERP. Psychophysiology 23: 695–703. 3823345 10.1111/j.1469-8986.1986.tb00696.x

[47] Eimer M (1997) An Event-related Potential (ERP) Study of Transient and Sustained Visual Attention to Color and Form. Biol Psychol 44: 143–60. 9043651 10.1016/s0301-0511(96)05217-9

[48] Wijers AA, Mulder G, Okita T, Mulder LJM, Scheffers MK (1989) Attention to Color: An Analysis of Selection, Controlled Search, and Motor Activation, Using Event-Related Potentials. Psychophysiology 26: 89–109. 2922460 10.1111/j.1469-8986.1989.tb03137.x

[49] Ito TA, Bartholow BD (2009) The Neural Correlates of Race. Trends Cogn Sci 13: 524–531. 19896410 10.1016/j.tics.2009.10.002 PMC2796452

[50] Eagly AH, Mladinic A (1994) Are People Prejudiced Against Women? Some Answers From Research on Attitudes, Gender Stereotypes, and Judgments of Competence. Eur Rev Soc Psychol 5: 1–35.

[51] Rudman LA, Goodwin SA (2004) Gender Differences in Automatic In-Group Bias: Why Do Women Like Women More Than Men Like Men? J Pers Soc Psychol 87: 494–509. 15491274 10.1037/0022-3514.87.4.494

[52] Barber HA, Otten LJ, Kousta ST, Vigliocco G (2013) Concreteness in Word Processing: ERP and Behavioral Effects in a Lexical Decision Task. Brain Lang 125: 47–53. 23454073 10.1016/j.bandl.2013.01.005

[53] West WC, Holcomb PJ (2000) Imaginal, Semantic, and Surface-level Processing of Concrete and Abstract Words: An Electrophysiological Investigation. J Cogn Neurosci 12: 1024–1037. 11177422 10.1162/08989290051137558

[54] Berlad I, Pratt H (1995) P300 in Response to the Subject's Own Name. Electroencephalogr Clin Neurophysiol 96: 472–4. 7555920 10.1016/0168-5597(95)00116-a

[55] Ninomiya H, Onitsuka T, Chen C-H, Sato E, Tashiro N (1998) P300 in Response to the Subject's Own Face. Psychiatry Clin Neurosci 52: 519–522. 10215014 10.1046/j.1440-1819.1998.00445.x

[56] Gray HM, Ambady N, Lowenthal WT, Deldin P (2004) P300 as an Index of Attention to Self-relevant Stimuli. J Exp Soc Psychol 40: 216–224.

[57] Miyakoshi M, Nomura M, Ohira H (2007) An ERP Study on Self-relevant Object Recognition. Brain Cogn 63: 182–9. 17223240 10.1016/j.bandc.2006.12.001

[58] Tacikowski P, Nowicka A (2010) Allocation of Attention to Self-name and Self-face: An ERP Study. Biol Psychol 84: 318–324. 20298741 10.1016/j.biopsycho.2010.03.009

[59] Osterhout L, Bersick M, McLaughlin J (1997) Brain Potentials Reflect Violations of Gender Stereotypes. Mem Cognit 25: 273–285. 10.3758/bf03211283 9184479

[60] Schevernels H, Krebs RM, Santens P, Woldorff MG, Boehler CN (2013) Task Preparation Processes Related to Reward Prediction Precede Those Related to Task-difficulty Expectation. Neuroimage 84C: 639–647. 10.1016/j.neuroimage.2013.09.039 PMC3863725 24064071

[61] Kimura M, Takeda Y (2013) Task Difficulty Affects the Predictive Process Indexed by Visual Mismatch Negativity. Front Hum Neurosci 7: 267. 23781189 10.3389/fnhum.2013.00267 PMC3679470

[62] Fouriezos G, Bielajew C, Pagotto W (1990) Task-Difficulty Increases Thresholds of Rewarding Brain-Stimulation. Behav Brain Res 37: 1–7. 2310490 10.1016/0166-4328(90)90066-n

[63] Lingnau A, Petris S (2013) Action Understanding Within and Outside the Motor System: The Role of Task Difficulty. Cereb Cortex 23: 1342–50. 22617849 10.1093/cercor/bhs112

[64] Kaczkurkin AN (2013) The Effect of Manipulating Task Difficulty on Error-related Negativity in Individuals with Obsessive-compulsive Symptoms. Biol Psychol 93: 122–31. 23318942 10.1016/j.biopsycho.2013.01.001

[65] Hughes ME, Johnston PJ, Fulham WR, Budd TW, Michie PT (2013) Stop-signal Task Difficulty and the Right Inferior Frontal Gyrus. Behav Brain Res 256: 205–13. 23973765 10.1016/j.bbr.2013.08.026

[66] Kremlacek J, Kuba M, Kubova Z, Langrova J, Szanyi J, et al (2013) Visual Mismatch Negativity in the Dorsal Stream is Independent of Concurrent Visual Task Difficulty. Front Hum Neurosci 7: 411. 23908621 10.3389/fnhum.2013.00411 PMC3726860

[67] Verner M, Herrmann MJ, Troche SJ, Roebers CM, Rammsayer TH (2013) Cortical Oxygen Consumption in Mental Arithmetic as a Function of Task Difficulty: A Near-infrared Spectroscopy Approach. Front Hum Neurosci 7: 217. 23734120 10.3389/fnhum.2013.00217 PMC3660659

[68] Green N, Bogacz R, Huebl J, Beyer AK, Kuhn AA, et al (2013) Reduction of Influence of Task Difficulty on Perceptual Decision Making by STN Deep Brain Stimulation. Curr Biol 23: 1681–4. 23932401 10.1016/j.cub.2013.07.001

[69] Merola JL, Liederman J (1990) The Effect of Task Difficulty upon the Extent to Which Performance Benefits from Between-hemisphere Division of Inputs. Int J Neurosci 51: 35–44. 2265907 10.3109/00207459009000506

[70] Sedikides C, Olsen N, Reis HT (1993) Relationships as Natural Categories. J Pers Soc Psychol 64: 71–82.

[71] Bretherton I (2010) Fathers in Attachment Theory and Research: A Review. Early Child Dev Care 180: 9–23.

[72] Tottenham N (2012) Human Amygdala Development in the Absence of Species-expected Caregiving. Dev Psychobiol 54: 598–611. 22714586 10.1002/dev.20531 PMC3404246

