# Supplementary material for: The Neural Response to Maternal Stimuli: An ERP Study
Source: PLoS One. 2014 Nov 6;9(11):e111391. doi: 10.1371/journal.pone.0111391 (PMC4222870; doi:10.1371/journal.pone.0111391)
Supplement: Text S1 — Response-locked ERP analysis for mother and others words with Go response. (DOCX) [file pone.0111391.s001.docx]

**Text S1:** Response-locked ERP analysis for *mother* and *others* words with Go response

We analyzed the response-locked ERPs to directly rule out the potential concern that the late positive potential (LPP) difference between *mother + good* and *mother + bad* conditions in the stimuli-locked ERP waves (see the manuscript) was the result of the different reaction speed, rather than the difference in terms of the evaluative process. If the different reaction speed of identical key-pressing response led to the LPP difference, there would be no significant difference prior to the response in the response-locked ERP waves between *mother + good* and *mother + bad* condition. On the contrary, if the evaluative inconsistency led to the enhanced LPP in *mother + bad* condition in contrast to *mother + good* condition, the amplitude of the ERP wave prior to key-pressing in the response-locked ERPs would be larger in *mother + bad* condition than in *mother + good* condition. In addition, since evaluative inconsistency did not occurs in the evaluating of others, there would be no significant difference in the amplitude of the ERP wave prior to key-pressing in the response-locked ERPs between *others + bad* and *others + good* conditions.

The onset of the key-pressing response was set at zero point. The continuous EEG data was epoched into periods of 1400 ms including a 800-ms post-response period and a 600-ms pre-response period. The period between 500-700ms after the key-pressing was set as baseline. We selected this period as baseline for two reasons. First, mental process was expected to be similar between conditions during this period. In detail, the fixation was presented on the screen after the key-pressing response, and participants were instructed to watch the fixation and wait for the next stimuli word. Second, the influence of key-pressing on electrophysiological signals, particularly, the motor potential (MP), was expected to disappear during this period (see Fabiani M, Gratton G & Coles M G H, 2000). Thereby the ERP wave of this period was comparable between *mother + good* and *mother + bad* conditions, which meets the requirement of baseline correction.

After the baseline correction, trials with artifacts due to eye blinks, amplifier clipping, and burst of electromyographic (EMG) activity exceeding ±100 μV were excluded before averaging. In addition, data of trials with incorrect response was also excluded from final averaging. The ERPs for category words (*mother* or *others*) with Go response from the four blocks were averaged separately. Finally, two types of ERPs for each category words were obtained.

The grand averaged ERPs to *mother* or *others* words from Go trials are shown in Figure S1a and Figure S1b, respectively. To compare the brain activity prior to the response, the mean amplitude from -200 to 0 ms over the centro-parietal area (12 sites: C3, CZ, C4, CP3, CPZ, CP4, P3, PZ, P4, PO3, POZ and PO4) was measured and submitted into a four-way ANOVA (target category (*mother* vs. *others*) × valence (*good* vs. *bad*) × Anterior-Posterior (C vs. CP vs. P vs. PO) × Laterality (left vs. midline vs. right). The results showed a significant there-way interaction (target category × valence × Laterality). *F* _(1, 22)_ = 6.59, *p* = .003, *partial η^2^* = 0.23. Further test showed a significant interaction between target category and valence over midline centroparietal area, *F* _(1, 22)_ = 6.79, *p* = .016, *partial η^2^* = 0.24. Simple test revealed that the ERP wave in *mother + bad* condition (*M* = 9.51 μV) was larger than that in *mother + good* condition (*M* = 7.25 μV), *F* _(1, 22)_ = 9.84, *p* = .005, *partial η^2^* = 0.31. In contrast, no significant difference was found between *others + bad* (*M* = 7.82 μV) and *others + good* conditions (*M* = 8.04 μV), *F* _(1, 22)_ = 0.19, *p* = .668, *partial η^2^* = 0.01. These results confirmed our anticipation that the evaluative inconsistency led to the augment of ERP waves prior to motor response in *mother + bad* compared with *mother + good* condition. More relevantly, it suggested that the late positive potential (LPP) amplitude difference between *mother + bad* and *mother + good* condition (see the manuscript) could not be interpreted as the result of behavioral response difference. Rather, we believed that the LPP findings reflected the affective processing of *mother* words.

**Figure Legend**

*Figure S1.* Grand averaged ERPs for target category words. The light gray shaded areas indicate the time window for the detection of the LPP component.

**Reference**

Fabiani M, Grantton G & Coles M G H (2000). Event-related potentials. In Cacioppo T, Taasnary L G & Berntson G G. (Ed.) *Handbook of Psychophysiology* (pp. 53-84). Cambridge: Cambridge University Press.
