# Supplementary figures and images for: The Neural Response to Maternal Stimuli: An ERP Study
Source: PLoS One. 2014 Nov 6;9(11):e111391. doi: 10.1371/journal.pone.0111391 (PMC4222870; doi:10.1371/journal.pone.0111391)

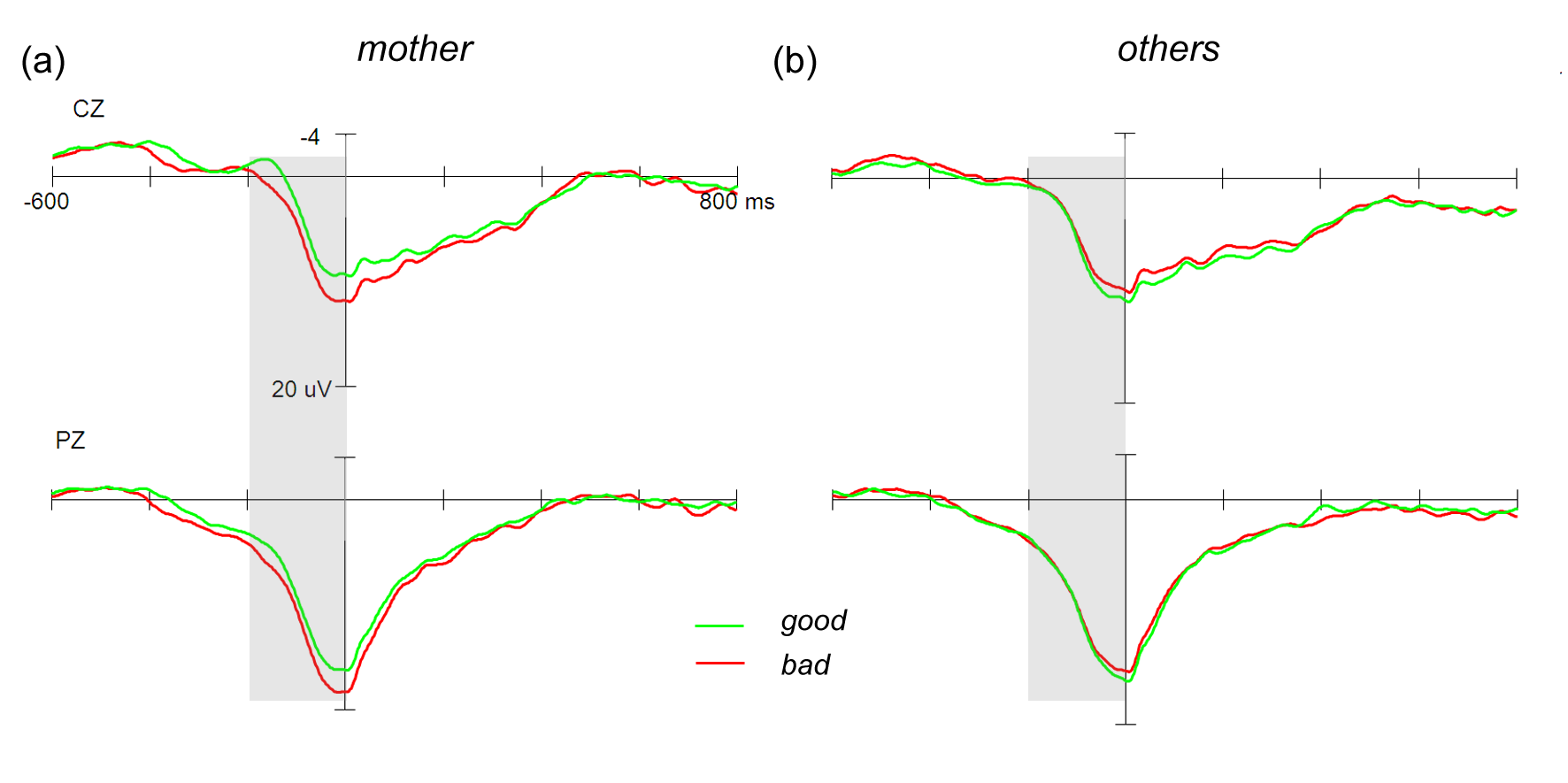

Supplement: Figure S1 — Grand averaged ERPs for target category words. The light gray shaded areas indicate the time window for the detection of the LPP component. (TIF) [file pone.0111391.s002.tif]
